# Outcomes of micropulse cyclophotocoagulation for refractory glaucoma in children

**DOI:** 10.1007/s10384-026-01354-z

**Published:** 2026-04-16

**Authors:** Tomoyo Yoshida, Tadashi Yokoi, Hazuki Anzai, Ayaka Nishida, Sachiko Nishina

**Affiliations:** Division of Ophthalmology, National Center for Child Health and Development, 2-10-1 O-kura, Setagaya-ku, Tokyo, 157-8535 Japan

**Keywords:** Micropulse transscleral cyclophotocoagulation, Pediatric glaucoma, Refractory glaucoma

## Abstract

**Purpose:**

To evaluate the outcomes of micropulse transscleral cyclophotocoagulation (MP-CPC) for refractory glaucoma in children.

**Study design:**

Retrospective study.

**Methods:**

We evaluated the data from children with refractory glaucoma who underwent MP-CPC from July 2022 to December 2024 at the National Center for Child Health and Development and who were followed up for at least 3 months. The retrospective analysis included age; glaucoma type; history of glaucoma surgery; intraocular pressure (IOP) at baseline and at 1, 3, and 6 months postoperatively; medication score administered before and after surgery; visual acuity; and complications.

**Results:**

Twenty-nine eyes of 24 patients (average age, 101.3 ± 68.1 months) were included in this study. The most common types of glaucoma were glaucoma associated with nonacquired ocular anomalies and glaucoma after cataract surgery, accounting for 11 eyes (37.9%). The average number of glaucoma surgeries performed before treatment was 1.9 ± 1.6. The average follow-up period was 16.1 ± 7.9 months. Using a mixed-effects model, the mean IOP decreased by −9.8 mm Hg at 1 month (P <.01), −7.5 mm Hg at 3 months (P <.01), and −7.4 mm Hg at 6 months (P <.01). The median medication score (interquartile range) decreased from 5 (4–6) preoperatively to 4 (3–5) at 1, 3, and 6 months postoperatively. After undergoing the first MP-CPC, 19 eyes (65.5%) required reoperation for glaucoma. Eleven eyes (37.9%) required reoperation within 6 months postoperatively, and 17 eyes (58.6%) required reoperation within 1 year.

**Conclusion:**

MP-CPC effectively reduces IOP in children with refractory glaucoma without causing visual impairment or serious complications.

## Introduction

Pediatric glaucoma is one of the most challenging ocular diseases to treat. In particular, glaucoma associated with congenital anterior-segment structural abnormalities or glaucoma that develops secondary to other eye diseases such as cataracts, retinal detachment, or retinopathy of prematurity presents additional anatomic and pathophysiologic complexities, making the development of appropriate treatment strategies extremely difficult [1]. Even if a temporary reduction in intraocular pressure (IOP) is achieved, elevated IOP can recur during long-term follow-up, often requiring multiple surgical interventions. Conventional treatments such as trabeculotomy, trabeculectomy, and various device implantation procedures (Ahmed valve, New World Medical; Baerveldt tube, Johnson and Johnson Surgical Vision) can suppress the IOP to a certain level in the short term [2–5], but they often have limitations in maintaining long-term visual function owing to postoperative complications and the strong scar formation response characteristic of children. Thus, when treating pediatric glaucoma, 1 surgical procedure or treatment modality is insufficient, and a long-term, multifaceted, staged treatment plan is required. The establishment of a treatment method that can safely maintain stable visual acuity (VA) and the visual field over the long term remains an important challenge.

In recent years, micropulse transscleral cyclophotocoagulation (MP-CPC) using micropulse waves has emerged as a new treatment option [6]. Conventional continuous wave transscleral cyclophotocoagulation (CW-CPC) has demonstrated high efficacy in reducing IOP; however, because it carries a high risk of severe complications such as significant tissue damage, vision loss, and ocular atrophy, it is typically reserved for endstage cases or those unresponsive to other treatments. In contrast, MP-CPC delivers energy in intermittent pulses, thereby minimizing thermal damage to the ciliary body while suppressing aqueous humor production and promoting uveoscleral outflow to reduce the IOP. As a result, this minimally invasive treatment, which minimizes postoperative inflammation and pain while preserving visual function and ocular morphology, has been primarily applied to adult glaucoma [7–9].

Reports on the efficacy and safety of MP-CPC in pediatric glaucoma remain insufficient [10–14]. Considering the anatomic and physiologic characteristics unique to children, it is necessary to evaluate risks specific to this population, including postoperative inflammatory responses, scar formation, the effects of multiple procedures on long-term outcomes, and the impact on ocular growth.

The aim of this study was to retrospectively analyze cases of refractory pediatric glaucoma treated with MP-CPC to clarify its therapeutic efficacy and the frequency of postoperative complications. We also describe patients who underwent multiple treatments during the study.

## Patients and methods

This retrospective, uncontrolled study included 29 eyes of 24 patients with refractory pediatric glaucoma who underwent MP-CPC between July 2022 and December 2024 at the National Center for Child Health and Development. The inclusion criteria were a minimal follow-up period of 3 months postoperatively and the ability to undergo measurement of IOP using tonometry.

All the MP-CPC procedures were performed under general anesthesia. MP-CPC was performed by use of the Cyclo G6 Laser (Iridex Corporation) in combination with the MicroPulse P3 probe. The laser settings included a power of 2500 mW and a duty cycle of 31.3%, with on and off durations of 0.5 msec and 1.1 msec, respectively. During the initial treatment, laser energy was applied for 160 seconds, ie, 80 seconds each to the superior and inferior hemispheres. In subsequent treatments, the total duration was extended up to 320 seconds, ie, 160 seconds/hemisphere, depending on the clinical condition, such as those with a recurrence within 3 months or with an IOP that increased again above 35 mm Hg or those undergoing MP-CPC 3 or more times.

The following clinical parameters were collected and analyzed: age at the time of MP-CPC, sex, presence of systemic comorbidities, type of glaucoma, number of glaucoma surgeries before MP-CPC, and length of follow-up. The follow-up period was defined as the time from the date of the primary surgery to the date of the final follow-up visit. When the IOP rose again, follow-up observation was continued in cases of retreatment with MP-CPC, and observation was terminated in cases in which another glaucoma surgery was performed. The IOP was measured preoperatively and at 1, 3, and 6 months postoperatively by use of the following tonometers depending on the patient’s condition and cooperation: the iCare rebound tonometer (iCare USA), Perkins applanation tonometer (Haag-Streit), Goldmann applanation tonometer (Haag-Streit), or Schiøtz tonometer (Handaya). The IOP was measured primarily by use of Goldmann applanation tonometry during outpatient examinations. When that measurement was not feasible, particularly in uncooperative pediatric patients, the IOP values obtained with the iCare rebound tonometer were used. For the preoperative assessment, if measurement using Goldmann tonometry was possible in the outpatient setting, those values were adopted. Otherwise, the mean IOPs measured with the Schiøtz and Perkins tonometers under general anesthesia were used. These measurements were treated as equivalent for analysis, consistent with previous pediatric glaucoma studies.

The best-corrected VA (BCVA) and antiglaucoma medication score were evaluated preoperatively and at 3 months postoperatively. The postoperative complications, the number of additional surgical interventions, and the time to reoperation were also recorded. The antiglaucoma medication score was calculated to quantify the pharmacologic burden. Each single-agent eye drop was assigned 1 point, combination eye drops were assigned 2 points, and oral carbonic anhydrase inhibitors were assigned 2 points. The total score was compared preoperatively and postoperatively. The medication score was expressed as the median and interquartile range (IQR) values. A responder analysis was conducted to evaluate the treatment success rates. The responders were defined as those with a 20% or greater reduction in IOP and either a maintained or a reduced medication score used at each follow-up visit. Eyes that underwent additional glaucoma surgery during the follow-up period were excluded from the analyses of the postoperative medication score and responder rate at the corresponding time points.

### Statistical analysis

The longitudinal changes in the IOP were evaluated using a linear mixed-effects model to account for the within-patient correlation. Because only a small number of patients had data from both eyes, eye-level clustering was explored but not retained in the final model. Accordingly, a random intercept for each patient was included (IOP ~ Time + (1|Patient.ID)). Time was treated as a categorical variable to allow for nonlinear postoperative changes. The paired *t* test was used to compare the preoperative and postoperative BCVA.

Kaplan–Meier survival analysis was performed with time-to-reoperation as the primary endpoint to evaluate surgical success over time. The survival rate was defined as the proportion of eyes that did not experience surgical failure during the follow-up period. Failure was defined as the need for an additional glaucoma surgery, including repeated MP-CPC or other glaucoma procedures. Reoperation was indicated when the predefined IOP criteria were met: IOP ≥22 mm Hg on 2 consecutive visits or IOP ≥30 mm Hg confirmed to be consistent with increased ocular firmness on digital palpation. Eyes that maintained adequate IOP control without additional surgery were regarded as successful cases. We also performed sensitivity analysis using a standard success definition. Failure was defined as either (1) IOP >22 mm Hg requiring additional IOP-lowering medication or (2) failure to achieve IOP ≤21 mm Hg on 2 consecutive postoperative visits. The time to failure was evaluated by use of Kaplan–Meier analysis and log-rank testing. The median survival times and corresponding 95% CIs were reported.

Univariate analysis was conducted to identify factors associated with reoperation within 6 months of the first MP-CPC. Probability values less than 0.05 were considered significant.

All statistical analyses were performed with EZR (Saitama Medical Center, Jichi Medical University), which is a graphical user interface for R (The R Foundation for Statistical Computing).

### Ethical considerations

This study was conducted in accordance with the principles of the Declaration of Helsinki and was approved by the institutional review board of the National Center for Child Health and Development (approval number: 2023-013).

## Results

Table 1 shows the patient demographic and clinical data. Twenty-nine eyes of 24 patients (9 boys [37.5%], 15 girls [62.5%]; mean age, 101.3 ± 68.1 months) were included. Seven patients (29.2%) had systemic diseases, including developmental delay, autism, asthma, cleft palate, atrial septal defect, punctate chondrodysplasia, low spinal cone, syringomyelia, thoracic spinal canal stenosis, and epilepsy. The most common types of glaucoma were those that developed after cataract surgery and those associated with nonacquired ocular anomalies (11 eyes each, 37.9%), which includes aniridia (7 eyes) and Axenfeld-Rieger anomaly (1 eye). The average number of glaucoma surgeries performed before treatment was 1.9 ± 1.6, including trabeculotomy, trabeculectomy, and PreserFlo Microshunt (Santen Pharmaceutical) implantation. The average follow-up period was 16.1 ± 7.9 months.

**Table 1:** Baseline demographic and clinical data from 24 pediatric refractory glaucoma patients who underwent MP-CPC

Age, mo		101.3 ± 68.1
Sex	Male	9 (37.5%)
	Female	15 (62.5%)
Presence of systemic diseases		7 (29.2%)
Glaucoma diagnosis	Primary congenital glaucoma	5 (17.2%)
	Glaucoma associated with nonacquired ocular anomalies	11 (37.9%)
	Glaucoma associated with nonacquired systemic disease or syndrome	1 (3.5%)
	Glaucoma associated with acquired condition	1 (3.5%)
	Glaucoma following cataract surgery	11 (37.9%)
No. previous glaucoma surgeries		1.9 ± 1.6
Follow-up period, mo		16.1 ± 7.9

Table 2 shows the preoperative and postoperative ocular parameters with follow-up visits. In the longitudinal analysis using a linear mixed-effects model with time treated as a categorical predictor and a patient-level random intercept, the IOP decreased significantly at all postoperative time points when compared with baseline. The mean IOP had decreased by −9.8 mm Hg at 1 month (*P* < 0.01), −7.5 mm Hg at 3 months (*P* < 0.01), and −7.4 mm Hg at 6 months (*P* < 0.01). These results indicate a marked early postoperative IOP reduction followed by a modest rebound, whilst the IOP remained significantly lower than baseline throughout the 6-month observation period.

**Table 2:** Comparison of preoperative and postoperative ocular parameters after the first MP-CPC with follow-up visits

Index	Phase	Mean (SD)
IOP, mm Hg	Preoperative (n = 29)	30.1 ± 9.8
	1 month postoperative (n = 29)	20.0 ± 8.2
	3 months postoperative (n = 28)	22.5 ± 9.6
	6 months postoperative (n = 21)	21.8 ± 8.9
BCVA, logMAR	Preoperative (n = 23)	1.7 ± 0.3
	3 months postoperative (n = 23)	1.6 ± 0.3
Index	Phase	Median (IQR)
Medication score	Preoperative	5 (4–6)
	1 month postoperative (n = 29)	4 (3–5)
	3 months postoperative (n = 28)	4 (3–5)
	6 months postoperative (n = 21)	4 (3–5)

*logMAR* logarithm of the minimum angle of resolution

Among the patients who underwent a VA examination, the BCVA improved significantly (*P* < 0.05, Wilcoxon signed rank test).

The median (interquartile range [IQR]) medication score was 5 (4–6) preoperatively, 4 (3–5) at 1 month, 4 (3–5) at 3 months, and 4 (3–5) at 6 months postoperatively. On the basis of the responder definition (≥20% IOP reduction from baseline and maintenance of or reduction in the number of antiglaucoma medications), the success rates were 19/29 (65.5%) at 1 month, 13/28 (46.4%) at 3 months, and 9/21 (42.9%) at 6 months postoperatively.

During the study period, 19 eyes required at least 1 reoperation (total number of reoperations, 35; mean, 1.8 ± 1.1; range, 1–5). Of the 35 reoperations, 27 (77.1%) were MP-CPC procedures and eight (22.9%) were other glaucoma surgeries, including implantation of 5 PreserFlo Microshunts, 2 trabeculectomies, and 1 Ahmed glaucoma valve.

**Fig. 1:**
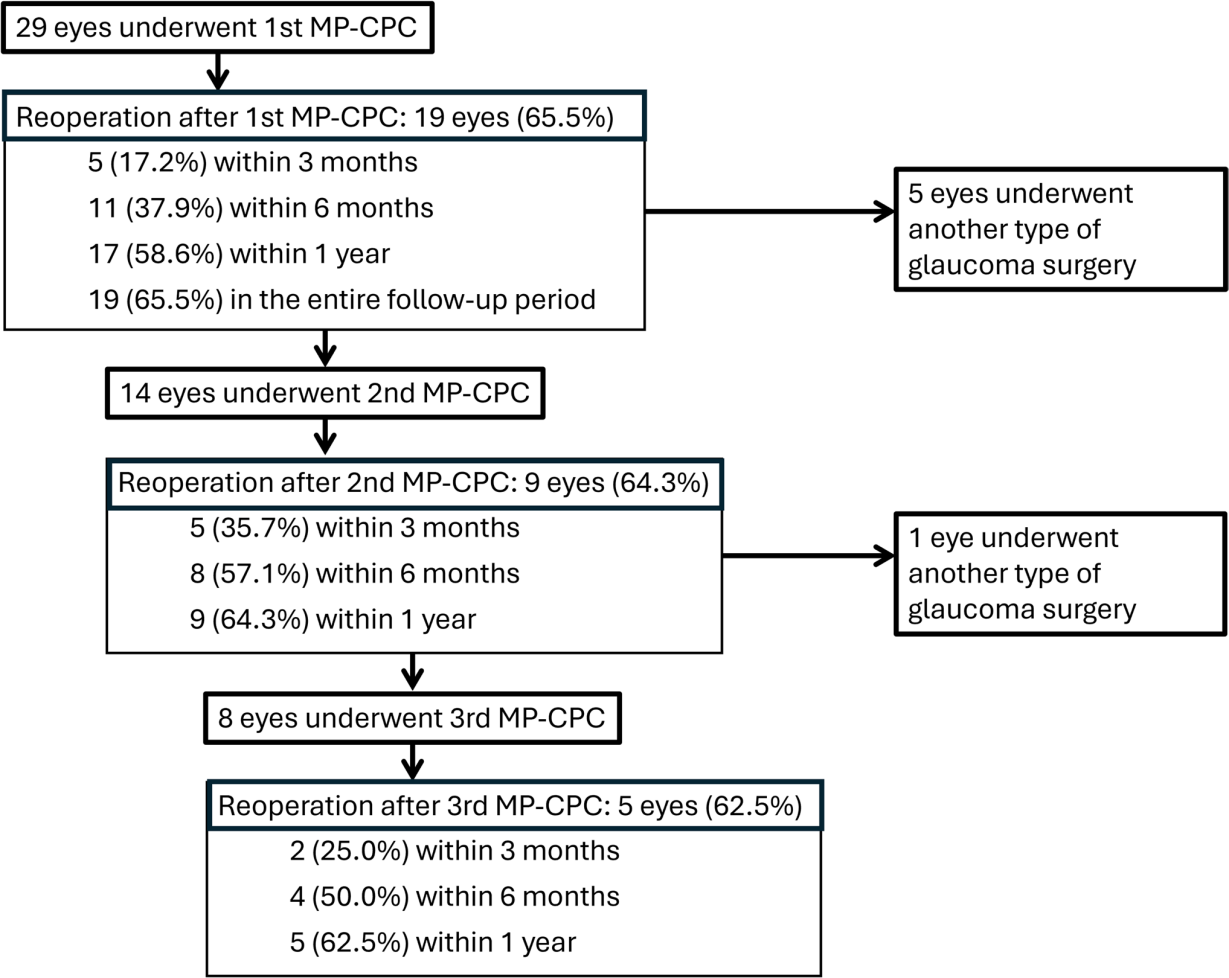
Flow diagram of reoperations following repeated MP-CPC treatments. The diagram illustrates the clinical course of 29 eyes that underwent initial MP-CPC, including the number of eyes requiring reoperation after the first, second, and third MP-CPC procedures. Reoperation was defined as a repeated MP-CPC or other glaucoma surgery owing to insufficient IOP control. The cumulative proportions of eyes requiring reoperation within 3 months, 6 months, and 1 year of each MP-CPC session are shown

Figure 1 shows the cumulative reoperation and success rates of the first, second, and third MP-CPC procedures. After the first MP-CPC of the 29 eyes, 5 eyes (17.2%) required reoperation within 3 months; 11 eyes (37.9%), within 6 months; and 17 eyes (58.6%), within 1 year.

Among the cases that required retreatment after the first MP procedure, 14 eyes underwent a second MP-CPC. Of these, 5 eyes (35.7%) had a recurrent IOP elevation within 3 months postoperatively; eight (57.1%), within 6 months postoperatively; and nine (64.3%), within 1 year postoperatively, necessitating retreatment.

Similarly, among the eyes that required retreatment after the second MP procedure, eight underwent a third MP-CPC. Of these, 2 eyes (25.0%) had a recurrent IOP elevation within 3 months postoperatively; four (50.0%), within 6 months postoperatively; and five, (62.5%) within 1 year postoperatively, necessitating retreatment.

**Fig. 2:**
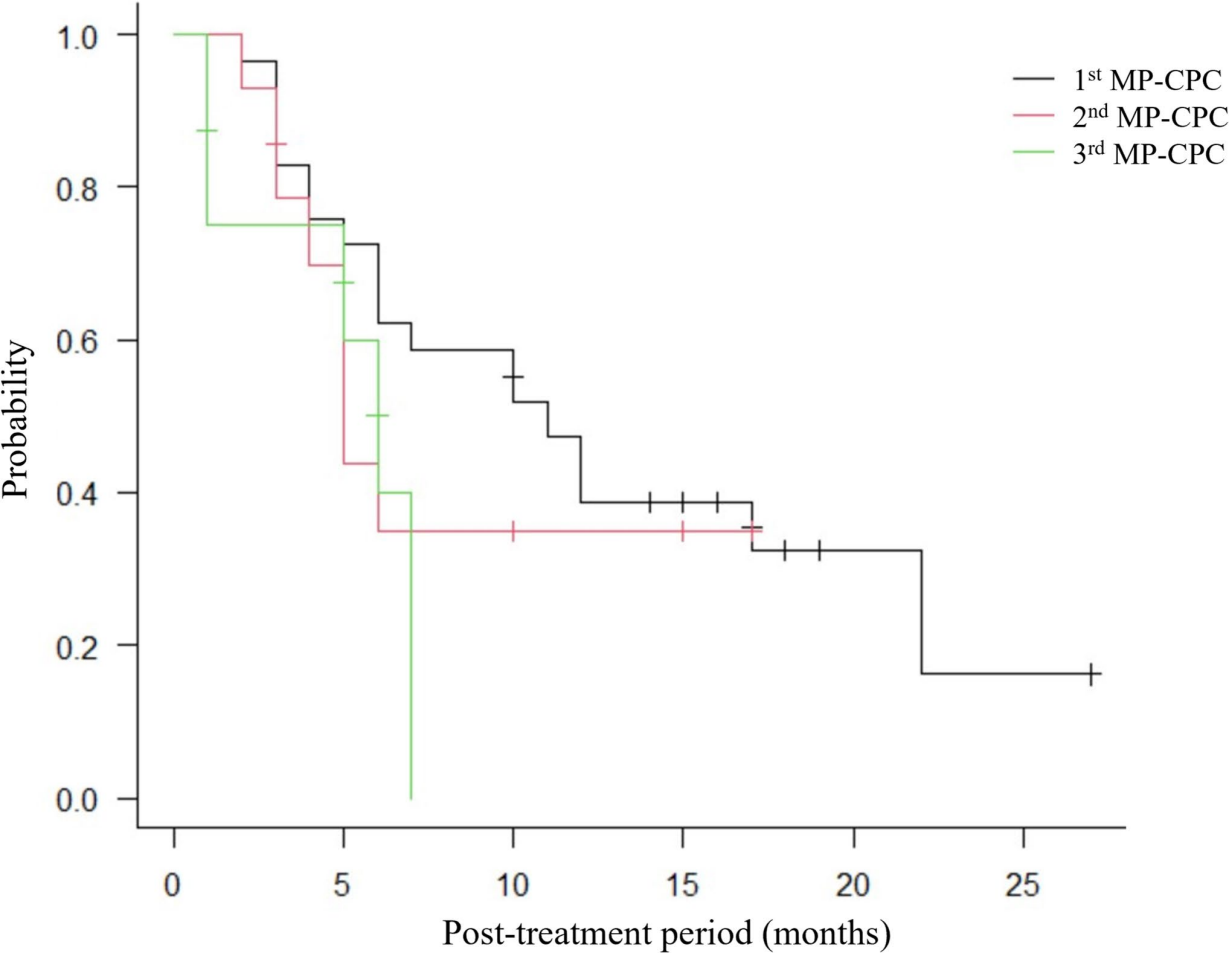
Kaplan–Meier survival curves of the first, second, and third MP-CPC procedures. No significant differences were observed in the efficacy of MP-CPC based on the surgical frequency (*P* = 0.276, log-rank test)

The Kaplan–Meier survival curves for the time to reoperation are shown in Fig. 2. The median survival times were 11 months (95% CI 6–22) for the first MP-CPC group, 5 months (95% CI 3–not applicable [NA]) for the second MP-CPC group, and 6 months (95% CI 1–NA) for the third MP-CPC group. The log-rank test indicated no significant differences among the 3 groups (*P* = 0.276). Using the standard success definition, failure-free survival was also compared among the groups. The median survival times were 6.0 months (95% CI 3–7), 2.5 months (95% CI 2–9), and 4.0 months (95% CI 1–NA) for the first, second, and third MP-CPC groups, respectively. No significant differences were detected on log-rank testing (*P* = 0.425).

Table 3 shows the frequency of complications following treatment. After the first MP-CPC, the most common adverse reaction was conjunctival hyperemia (6 eyes, 20.7%), followed by pain (5 eyes, 17.2%) and nausea (4 cases, 16.7%). No serious complications were observed, and all complications resolved within a few days.

**Table 3:** Frequency of complications after the first MP-CPC procedure

Ocular complication	No. (%) after 1 MP-CPC (n = 29)	No. (%) after 2 MP-CPCs (n = 14)	No. (%) after 3 MP-CPCs (n = 8)
No complications	10 (34.5)	9 (64.3)	3 (37.5)
Hyperemia	6 (20.7)	2 (14.3)	1 (12.5)
Pain	5 (17.2)	2 (14.3)	3 (37.5)
Inflammation	3 (10.3)	2 (14.3)	2 (25)
Brightness	3 (10.3)	1 (7.1)	1 (12.5)
Tearing	1 (3.4)	0	0
Systemic complication	No. (%) after 1 MP-CPC (n = 24)	No. (%) after 2 MP-CPC (n = 14)	No. (%) after 3 MP-CPCs (n = 8)
Nausea	4 (16.7)	0	0

Table 4 shows the results of univariate analysis of factors associated with reoperation within 6 months of the first MP-CPC. Age, history of glaucoma surgery, and presence of systemic diseases were not associated with early reoperation. The preoperative and 1-month IOPs were also not associated with early reoperation, but the 3-month IOP (odds ratio: 1.12; CI 1.01–1.25, *P* < 0.05) was associated with early reoperation. No association was seen between the preoperative and postoperative medication scores and early reoperation.

**Table 4:** Univariate analysis of factors associated with reoperation within 6 months of the first MP-CPC

Factor	OR	95% CI	*P* value
Age	0.998	0.987–1.01	0.762
History of glaucoma surgery	0.611	0.289–1.29	0.198
History of systemic diseases	0.444	0.0721–2.74	0.382
IOP			
Preoperative	1.01	0.931–1.09	0.872
1 month postoperative	1.10	0.986–1.23	0.089
3 months postoperative	1.12	1.01–1.25	0.037
Medication score			
Preoperative	0.877	0.477–1.61	0.672
3 months postoperative	1.69	0.972–2.950	0.063

*OR *odds ratio

Table 5 shows the results of univariate analysis of reoperation within 6 months of the first MP-CPC and the type of glaucoma. No association was seen between any glaucoma type and the need for reoperation. In addition, no significant difference was seen in the time until reoperation based on glaucoma type (*P* = 0.1961, Kruskal–Wallis rank-sum test).

**Table 5:** Univariate analysis of reoperation within 6 months and type of glaucoma

	Reoperated cases			
	No.	OR	95% CI	*P* value
Glaucoma type				
Primary congenital glaucoma	1	Reference		
Glaucoma associated with nonacquired ocular anomalies	4	2.29	0.185–28.2	0.519
Glaucoma associated with nonacquired systemic disease or syndrome	1	Inf	0-Inf	0.996
Glaucoma associated with acquired condition	1	Inf	0-Inf	0.996
Glaucoma following cataract surgery	4	2.29	0.185–28.2	0.519

*OR* odds ratio, *Inf* infinity

## Discussion

This retrospective study evaluated the efficacy and safety of MP-CPC for refractory pediatric glaucoma and identified significant IOP-lowering effects without severe complications that impair visual function. In previous reports summarizing the long-term outcomes of surgical treatments, the goniotomy success rates ranged from 30 to 90%; those for traditional trabeculotomy, from 24 to 97.5%; those for circumferential trabeculotomy, from 72 to 93%; those trabeculectomy, from 62.5 to 81.3%; and those for glaucoma drainage devices, from 80 to 90% [15]. Whilst these results were inferior to existing surgical procedures regarding long-term IOP control, the present treatment offers a unique option in that it can be repeated safely without compromising the surgical field.

Few reports have evaluated the treatment outcomes of MP-CPC therapy for pediatric glaucoma; however, all previous studies have reported significant reductions in IOP [10–14, 16]. When compared with reports with a similar study design, the current study showed a higher treatment success rate at 6 months than that reported by Balbaid and colleagues, but a slightly lower rate at 12 months [10]. Balbaid and colleagues reported that outcomes were better with older patients at the time of surgery, so differences in patient age may be contributory. However, Sivasubramanian and colleagues reported worse 1- and 6-month outcomes in patients with a higher average age than that of the current cases, suggesting that factors other than age may influence treatment outcomes [11]. In fact, the current univariate analysis found no association between age and the need for retreatment. Similar findings were observed for other clinical factors such as previous history of glaucoma surgery, disease type, preoperative IOP, and topical eye-drop scores, except for the postoperative IOP, which was not identified as a risk factor for retreatment. Previous reports also failed to identify an association between these factors and resurgery [17], suggesting that MP-CPC may be broadly applicable regardless of disease type or background, whilst also indicating that the risk of IOP recurrence exists to some extent regardless of disease type.

Regarding complications, no serious adverse events such as severe postoperative inflammation or visual loss were observed, confirming that this is a safe treatment method, consistent with previous reports [11]. Whilst visual loss after MP-CPC has been reported in adults [7], the vision improved in some pediatric cases, possibly attributable to improved IOP control or increased accuracy of testing owing to age-related developmental changes. When compared with reports of complications associated with CW-CPC in refractory pediatric glaucoma [12, 13, 18–20], MP-CPC demonstrated fewer complications and greater safety.

The current case series includes patients who underwent repeated MP-CPC procedures, and we also presented survival curves for the second and third MP-CPC procedures. Previous reports have indicated that the IOP-lowering effect of MP-CPC is sustained [9]. In refractory pediatric glaucoma, in which long-term disease progression is anticipated, complete cure after 1 surgery is difficult to achieve, and repeated treatments are often unavoidable. In this study, even in cases in which MP-CPC was performed 2 or more times, no significant difference was observed in the treatment success rate at 1 year, suggesting that the IOP-lowering effect persists. Additionally, no increase in complications was observed with an increased number of treatments, indicating that safety may be maintained despite multiple procedures. This is an important finding demonstrating that MP-CPC is a viable option for repeated use within a long-term treatment plan, representing a significant advantage in pediatric cases.

This retrospective study included a limited number of cases, so caution is required when generalizing the results. In addition, the observation period was relatively short, and long-term IOP control and the occurrence of late complications could not be adequately evaluated. Furthermore, the coexistence of multiple glaucoma subtypes introduced heterogeneity into the cohort, which may have influenced the treatment outcomes and limited the generalizability of the findings. Another limitation of this study is the heterogeneity in the treatment parameters, as the laser duration and total energy were adjusted according to the individual patient’s condition. Consequently, confounding resulting from the indication may have occurred since more severe or refractory cases tended to receive higher total energy or repeated MP-CPC sessions. In the future, it will be necessary to verify the efficacy and safety of MP-CPC over the long term through larger, prospective clinical studies.

In conclusion, MP-CPC is a minimally invasive treatment that can be safely administered multiple times without serious complications, demonstrating stable IOP-lowering effects in refractory pediatric glaucoma regardless of disease type. As a complementary treatment for cases that are difficult to manage with conventional therapies, MP-CPC may be a promising option in future pediatric glaucoma treatment strategies.
